# Cell membrane protein functionalization of nanoparticles as a new tumor-targeting strategy

**DOI:** 10.1186/s40169-019-0224-y

**Published:** 2019-03-15

**Authors:** Anna Pasto, Federica Giordano, Michael Evangelopoulos, Alberto Amadori, Ennio Tasciotti

**Affiliations:** 1 Veneto Institute of Oncology-IRCCS, Padua, Italy; 2 School of Medicine and Surgery, University of Milano-Bicocca, Monza, Italy; 3 Present Address: Center for Biomimetic Medicine, Houston Methodist Research Institute, 6670 Bertner Ave, Houston, TX 77030 USA; 4 Department of Surgery, Oncology and Gastroenterology, University of Padova, Padua, Italy; 5 Houston Methodist Orthopedics and Sports Medicine, Houston Methodist Hospital, Houston, TX USA

**Keywords:** Bioinspired, Biomimicry, Drug delivery, Nanoparticles, Cancer

## Abstract

Nanoparticles have seen considerable popularity as effective tools for drug delivery. However, non-specific targeting continues to remain a challenge. Recently, biomimetic nanoparticles have emerged as an innovative solution that exploits biologically-derived components to improve therapeutic potential. Specifically, cell membrane proteins extracted from various cells (i.e., leukocytes, erythrocytes, platelets, mesenchymal stem cells, cancer) have shown considerable promise in bestowing nanoparticles with increased circulation and targeting efficacy. Traditional nanoparticles can be detected and removed by the immune system which significantly hinders their clinical success. Biomimicry has been proposed as a promising approach to overcome these limitations. In this review, we highlight the current trends in biomimetic nanoparticles and describe how they are being used to increase their chemotherapeutic effect in cancer treatment.

## Background

Cancer is the second leading cause of death in the world with over 8 million deaths worldwide. Current treatment option range from surgery and radiation to chemo and hormone therapy [1]. Although technologies to combat cancer have made significant advances in eradicating the tumor mass and reducing metastasis, chemotherapy continues to remain the mainstay of tumor treatment. Unfortunately, chemotherapeutic administration often results in unwanted side effects and the development of drug resistance that can lead to cancer recurrence and metastatic dissemination. To mitigate the side effects, nanotechnology has been showcased as a versatile tool to increase drug potency and localize treatment [2–6]. Indeed, nanoparticles (NPs) are endowed with: (i) optimal drug loading properties [7], (ii) increased payload stability [8], prolonged circulation, (iii) enhanced permeability and retention (EPR), and (iv) biocompatibility via tunable chemical compositions [9, 10]. Despite these advantages, NP efficacy is often limited by the presence of biological barriers such as mononuclear phagocyte system (MPS), hemorheological forces [11] and endothelial vessel wall [12]. In an effort to overcome these hurdles, we and others have developed several classes of NPs characterized by different types of functions, responsive triggers, and surface modifications that contribute to increased their efficacy [13].

Initially, our group engineered multi-stage porous silicon NPs to mimic red blood cells and serve as a carrier to shuttle loaded nanoparticulates [14]. Strategically designed using fine-tuned size and shape parameters, these biocompatible [15] and biodegradable [10] multi-stage NPs leverage unique design properties [9] to enhance circulation and margination within vascular endothelia, thereby protecting the payload from various biological hurdles [16]. Nevertheless, targeted and localized delivery still require the use of various moieties (i.e., antibodies [17, 18], photosensitizers [19]) functionalized onto the NP surface, as well as the use of ‘smart’ materials [20] and lipid NPs [21]. Although substantial increases in targeting and efficacy were observed, MPS sequestration resulted in high accumulation in the liver and spleen, thereby hindering ideal localization at the tumor site. In addition, due to a number of biological barriers found within the body, it is paramount to strategically design a system that has the potential to circumvent these barriers sequentially while still maintaining the payload. For a thorough review in the biological barriers a nanoparticle is exposed to, we direct the reader to the following paper: [22].

**Fig. 1:**
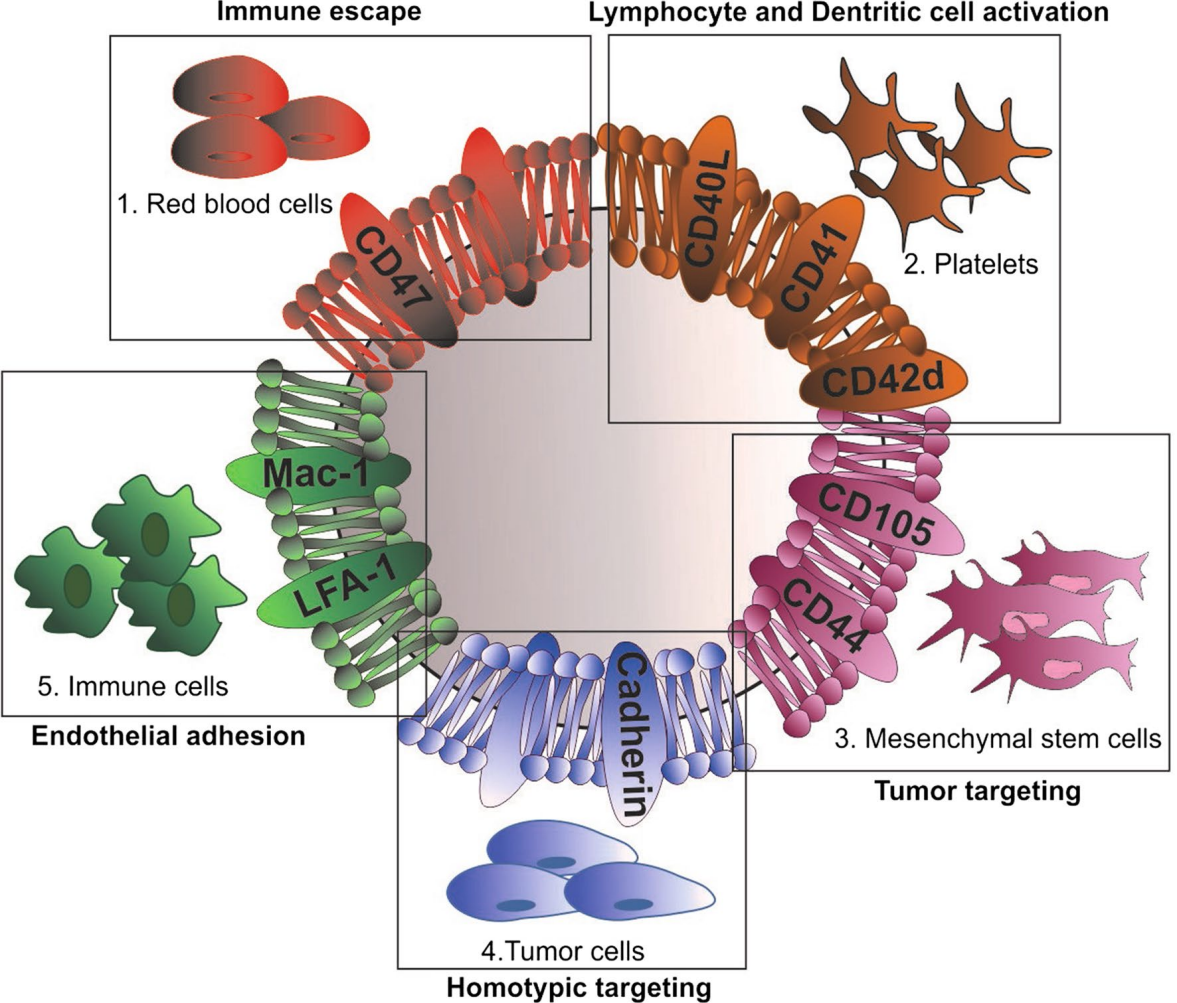
Schematic illustration of an empty biomimetic nanoparticle (NP) developed from the membrane sourced of different cells: platelets, red blood, mesenchymal, tumor and immune cells. Thanks to the cells of origin, NPs are endowed with specific features (in bold) that mediate their ability to escape the immune system, extravasate blood vessels and target tumor site. *LFA-1* lymphocyte function-associated antigen 1, *Mac-1* macrophage-1 antigen, *CD* Cluster of differentiation

In an effort to mitigate immunorecognition and sequestration by filtering organs, our group modified multi-stage NPs with a leukocyte-derived cell membrane shell as a strategy to bypass critical biological barriers [23]. Other groups have developed similar strategies consisting in using surface coatings derived from various cellular sources (i.e., mesenchymal stem cells [24], platelets [25], etc.), which transfer intrinsic cellular properties to a synthetic nanomaterial. Designed to leverage activated endothelia as a targeting method [26], this strategy has been shown to improve tumor targeting, provide prolonged circulation, and reduce immunoreactivity (Fig. 1).

The aim of this review is to highlight the various strategies in which biomimetic NPs are being used in cancer treatment. In addition, this review will cover the various cell sources employed for NP design and the intrinsic effects these cells provide in tumor targeting.

## Main text

### Source of cells for biomimetic nanoparticles

#### 1. Red blood cells

Due to unique biological properties such as prolonged blood circulation time, lack of organelles (i.e., nucleus), and abundance in the body, red blood cells (RBCs) represent the most convenient cell membrane protein source to produce cell-based NPs. In addition, thanks to the expression of specific inhibitory proteins such as CD47, also known as the “do not eat me” signal, RBCs can easily escape immune system recognition, inhibiting macrophage-mediated phagocytosis [27]. Zhang and coworkers were pioneers in the use of RBC membranes to develop biomimetic NPs. Specifically, they combined PLGA NPs with RBC membranes purified from fresh RBCs. The resulting RBC-NPs were validated for their protein content and long-term stability features, demonstrating successful translocation of the associated RBC membrane proteins to the NP surface. Thanks to the presence of immunosuppressive proteins on the RBC membrane (i.e., CD47), RBC-NPs showcased higher circulation half-life with significant retention in the blood and decreased macrophage uptake compared to conventional polyethylene glycol (PEG)-functionalized lipid-polymer hybrid nanoparticles (PEG-NPs). Overall, RBC-NPs resulted in higher structural rigidity, increased stability, and superior cargo encapsulation and delivery compared to uncoated NPs [28]. Further assessment of this technology in a lymphoma tumor murine model demonstrated the efficient delivery of doxorubicin (DOX) to tumor sites, leading to significant tumor growth inhibition while demonstrating positive immunocompatibility and safety relative to free drug [29].

Similarly, Su et al. formulated paclitaxel-loaded NPs using a polymeric core and a hydrophilic RBC vesicle shell (called RVPNs) that were co-administrated with the tumor-penetrating peptide, iRGD, to enhance antitumor therapy [30]. The authors demonstrated the advantages of the prolonged circulation of RVPNs and the tumor-penetration properties of iRGD in a murine breast cancer model. This strategy displayed remarkably higher retention of paclitaxel in the blood compared to conventional paclitaxel-loaded NPs. Specifically, RVPNs and iRGD achieved 90% tumor growth inhibition. In addition, this strategy showed positive results in the treatment of metastasis, exhibiting a 95% reduction of lung metastasis and substantially lower hematological toxicity compared to uncoated NPs, NPs/iRGD, or RVPNs alone [30].

#### 2. Platelets

Recently, platelets have also garnered significant attention as a source for biomimetic NPs. Derived from the bone marrow, these enucleated cells are involved in hemostasis, clotting, inflammation, as well as tissue repair [31]. Several studies have also demonstrated that platelets play a crucial role in carcinogenesis [32, 33]. Indeed, inflammation occurring during neoplastic progression recalls platelets to the tumor site, stimulating tumor angiogenesis. In addition, platelets sustain tumor cell extravasation and the survival of circulating tumor cells in the bloodstream [33], thus favoring metastatic spreading.

Taking advantage of the interactions between platelets and tumor cells, and thanks to their physical and biochemical properties such as discoidal shape and flexibility, biomimetic platelet-like NPs have been exploited for targeted drug delivery [34]. Li et al. produced silica (Si) NPs coated with membranes isolated from activated platelets (PMDV-coated Si particles) and functionalized with tumor necrosis factor (TNF)-related apoptosis inducing ligand (TRAIL) [35]. PMVD-coated Si-NPs were shown to express most of the platelet surface proteins (i.e., CD41, CD42b and CD61) and glycans relevant for targeting circulating tumor cells (CTCs) and escaping phagocytosis. Indeed, evaluation of a variety of cancer-bearing murine models (i.e., human breast cancer, colon cancer, and a syngeneic metastatic colon cancer and melanoma mouse model) demonstrated that TRAIL-conjugated PMDV-Si particles were able to efficiently target CTCs in lung vasculature and to dramatically decrease lung metastases compared to untreated mice, empty PMDV-coated Si particles, and soluble TRAIL. In addition, despite TRAIL is associated with an increase in liver toxicity, this strategy exhibited no substantial effect on hepatic apoptosis following a 24 h treatment.

A similar approach was used by Hu et al. that developed platelet membrane (PM)—coated core–shell nanovesicles (called PM-NVs) loaded with two anticancer components: TRAIL and DOX. The administration of PM-NVs in a breast cancer mouse model demonstrated NP accumulation at the tumor site and efficient delivery of TRAIL toward cancer cell membrane, resulting in the activation of the extrinsic apoptosis signaling pathway. Moreover, thanks to their acid-responsive encapsulation matrix, the PM-NVs were better digested after endocytosis, thus enhancing DOX intracellular accumulation. This resulted in the inhibition of tumor growth and a reduction in lung metastasis [36]. The same group, recently, exploited a combined strategy of two nanocarriers to enhance antitumor activity [37]. The first one was a signal transmission nanocarrier (NC_A_) functionalized with an RGD peptide to specifically target integrins (i.e., ανβ3) within tumor blood vessels. The second was a biomimetic nanocarrier (NC_B_) made of integrated platelet membranes and loaded with paclitaxel (PTX). The authors showed that most of the proteins expressed on the platelet membranes, such as CD36, CD42d, P-Selectin, and CD40L were successfully transferred onto the NP surface. This transfer mediated NP accumulation at the tumor site, reduced blood vessel inflammation and increased drug accumulation and treatment efficacy in the tumor cells [37]. Thanks to the specific affinity between P-selectin, transferred onto the NP surface, and CD44, overexpressed on the tumor cells, the authors demonstrated that their platelet membrane-coated biomimetic nanocarriers (PM-NPs) were endowed with the capability to target bone microenvironment and myeloma cells. PM-NPs showed high internalization, high tumor targeting, enhanced intracellular drug release, and decrease in off-target effects. Overall, compared to traditional active targeting, platelet-derived coating strategies increased selective bone targeting, as well as cytotoxicity against myeloma cells and the efficient thrombus dissolution in an in vivo multiple myeloma mouse model [38].

#### 3. Mesenchymal stem cells

It has been largely accepted that most current anti-tumor therapies fail as a result of a small cell population (referred as cancer stem cells, CSCs) responsible for tumor progression, tumor maintenance and metastases formation. CSCs have been identified and described in almost all types of cancer (i.e., prostate [39, 40], lung [41], colon [42], pancreatic [43], gastric [44], breast [45], glioma [46] and ovarian cancer [47, 48]). To date, no treatment has been successful in destroying or reducing CSCs due to their intrinsic properties (i.e., drug-resistance and ability to enter a quiescence state) and the lack of a universal marker for their identification.

Mesenchymal stem cells (MSCs) are multipotent stem cells endowed with the ability to differentiate into osteoblasts, adipocytes, chondroblasts, fibroblasts and pericytes. Stimulated by microenvironmental factors such as hypoxia, ligands of Toll-like receptors or cytokine gradients within the extracellular matrix, MSCs migrate to injured and inflamed sites. With cancer being considered as a disease of chronic inflammation, it’s appropriate that the last decade has seen a considerable rise in MSCs used as a therapeutic tool in tumor treatment [19, 49].

In a biomimetic strategy employing MSCs, Timaner et al. developed nanoghosts (NGs) derived from the cytoplasmic membranes of MSCs. MSC-NGs retained the ability of MSCs to target inflamed endothelium, allowing safe and effective targeted delivery of their payload both in vitro and in vivo using a prostatic mouse cancer model. In addition, the authors demonstrated that MSC-NGs accumulated in proximity of CSCs within the tumor mass. The membrane proteins expressed on the NG surface mediated cell-to-cell interaction with CSCs [50], thus suggesting their potential role in eradicating this CSC subset.

A similar approach was used by Machluf and coworkers that resulted in a significantly higher accumulation of MSC-derived NGs within the tumor as compared to smooth muscle cell-derived NGs (used as a non-mesenchymal control). MSC-NGs that retained on their surface specific MSC integrins were shown to bind and fuse to the tumor cell surface and disrupt the plasma membrane, leading to a cytotoxic effect and tumor growth inhibition. The authors suggested that the anti-tumor effect observed using empty MSC-NGs was likely due to the interaction of NG with the different components of the tumor microenvironment [24]. Conversely, treatment with empty synthetic liposomes did not result in any interaction with the tumor microenvironment. Thus, it is fair to speculate that MSC-derived integrins may mediate MSC-NG interaction with tumor-infiltrating immune cells, blood vessel endothelium, and tumor-associated fibroblasts.

On the other hand, Gao et al. demonstrated that MSC-biomimetic NPs were endowed MSC-derived hypoimmunogenicity due to different molecular recognition moieties. Indeed, bone marrow derived MSC membrane-coated gelatin nanogels (SCMGs) showed reduced uptake by the MPS, reduced clearance by the MPS and reduced internalization by filtration organs (such as spleen, kidney, liver and lung) [51]. As a result, SCMGs displayed longer blood circulation time, higher in vitro targeting and increased in vivo accumulation within the tumor site, compared to uncoated nanogels. All together, these results suggest the use of MSC-derived biomimetic NPs as a promising tool for anti-cancer therapies by exploiting the unique tropism of MSCs to sites of inflammation.

#### 4. Tumor cells

Among the various camouflage strategies, cancer cell-derived biomimetic strategies have been considered as a promising option for the surface functionalization of NPs with cell membrane, due to the intrinsic homotypic aggregation properties of cancer cells and their ability to escape immunorecognition. Fang et al. previously demonstrated that cancer cell membrane-coated NPs (CCNPs) improved cancer targeting due to homotypic interaction [52]. In particular, the authors developed PLGA NPs coated with membrane proteins extracted from different melanoma cell lines. Due to the expression of similar tumor proteins, CCNPs showed a 40- and 20-fold increase in tumor uptake compared to RBC-coated NPs and uncoated PLGA, respectively. In a follow-up study, the authors coupled CCNPs with monophosphoryl lipid A (MPLA), a U.S. Food and Drug Administration-approved lipopolysaccharide-derivative that binds Toll-like receptor-4 [52]. They demonstrated that functionalized CCNPs presented the capability to deliver tumor antigens and induce dendritic cell (DC) maturation. Indeed, the incorporation of MPLA into CCNPs was associated with a significant up-regulation of DC maturation markers (CD40, CD80, and CD86), that facilitate tumor-specific immune response through the increase of antigen presentation and the interaction with lymphocytes, that leads to increase IFNγ secretion.

Chen et al. developed biomimetic NPs by fusing a cell membrane isolated from MCF-7 human breast cancer cells to an indocyanine green (ICG)/PLGA core [53]. Dubbed ICNPs, this strategy resulted in reduced liver and kidney uptake, as well as higher tumor accumulation due to cell affinity. In addition, ICNPs showed both efficient real-time dual-modal tumor imaging and enhanced photothermal therapy properties. Specifically, this strategy resulted in complete ablation of the tumor using a single dose of ICNPs.

Similarly, Sun et al. developed a strategy to exploit cancer cell membrane-coated NPs for targeted chemotherapy of homotypic tumors [54]. The authors developed paclitaxel-loaded polymeric nanoparticles (PPNs) using polycaprolactone (PCL) and pluronic copolymer F68 coated with 4T1 mammary breast cancer cell membranes (called CPPNs). In vitro studies demonstrated that CPPNs are endowed with high targeting specificity to the homotypic tumor cells and reduced macrophage uptake. These properties were confirmed in vivo in an orthotopic breast cancer and in a metastatic tumor mouse model. Indeed, in both murine models, administration of CPPNs was associated with an increase in NP accumulation both at the primary tumor site and at metastatic lesions. Interestingly, this strategy exhibited superior inhibition of tumor and lung metastasis growth when compared to paclitaxel alone.

#### 5. Immune cells

In recent years, immune cells have also been proven to be a promising source of coating for biomimetic NPs. Specifically, our group was the first to demonstrate the beneficial properties endowed to NPs when a leukocyte-derived cell source was used. This proof-of-concept was demonstrated with the functionalization of nanoporous silicon particles with cellular membranes isolated from leukocyte cells [23]. Dubbed leukolike vectors (LLVs), we successfully grafted over 300 proteins on the NP surface using isolated cell membrane patches, with over 50% being associated with cellular membrane [55]. Among them, we identified proteins that promote reduced MPS uptake (i.e., clusterin [56]), immune tolerance (i.e., CD47 and CD45 [57, 58]), and endothelium/tumor targeting (i.e., lymphocyte function-associated antigen 1 [LFA-1] and macrophage-1 antigen [Mac-1] [12, 23]). In addition, beyond simply targeting activated endothelium, we further dissected the specific role LLVs attain following functionalization. Specifically, we exhibited, following the transfer of membrane onto NPs, proteins grafted onto the NPs surface retained biological activity, resulting in molecular interplay between particles: cell that fostered increased drug targeting. To this end, LLVs were able to preferentially accumulate at sites of activated endothelium, resulting in the downstream phosphorylation of vascular endothelium (VE)-cadherin proteins [12]. This increase in phosphorylation led to a reduction of endothelial tight junctions, thereby facilitating the transport of a payload into the tumor microenvironment.

To further evaluate the role cell source may have in the process of transport and targeting, we next compared syngeneic- and xenogeneic-derived cell membrane with uncoated NPs [59]. This study revealed when a syngeneic cell source was employed as a biomimetic coating, macrophage avoidance was higher than xenogeneic-coated and uncoated NPs. This was further demonstrated with a delay in liver sequestration following in vivo administration, with an increase in blood circulation also being observed. Taken together, these results indicate that the presence of various proteins on the nanoparticle surface contributed to an increase in targeting, vascular permeability, and payload delivery.

A similar strategy for targeting activated endothelium was used in the design of protein-rich liposomal vesicles. Using leukocyte-derived cellular membranes, proteo-lipid vesicles (called leukosomes) were fabricated and found to successfully incorporate more than 340 distinct proteins with a majority being associated with plasma membrane [57]. As observed with LLVs, the presence of critical adhesion proteins (such as LFA-1 and Mac-1) within the leukosome bilayer resulted in significant accumulation of NPs at the activated endothelium. Specifically, using an inflamed ear murine model, leukosomes exhibited a sevenfold increase in accumulation when compared to bare liposome particles [57]. To further demonstrate leukosome natural tropism to inflamed endothelium, we evaluated their accumulation in a 4T1 breast cancer mouse model [58]. When compared to liposomes, leukosomes demonstrated a 16-fold increase in tumor tissue accumulation. In addition, a 4.5-fold increase was observed associated within tumor vessel lumen (i.e., vessel center) while a 14-fold increase over liposomes observed on the vessel wall (i.e., outer edge). Interestingly, these effects were largely due to the presence of both LFA-1 and CD45 on the leukosome surface as previously reported with LLVs. Indeed, blocking of either protein resulted in a 60% and 95% reduction in tumor accumulation, respectively. This strategy represents a viable option in the localized delivery of chemotherapeutics, with considerable promise also demonstrated as an imaging-guided or theranostic approach [58, 60].

Similar observations of anti-inflammatory effects were observed when biomimetic NPs were further functionalized with the α4β7 integrin commonly overexpressed in a T lymphocyte-subset that specializes in targeting gastrointestinal inflammation [61]. This strategy demonstrated that doping biomimetic NPs with this integrin resulted in increased association with the inflamed vessel area while bare NPs tended to diffuse from blood vasculature into the interstitial space, indicating poor adhesion with inflamed vasculature. Gene expression following treatment with doped NPs also indicated a significant reduction in anti- (i.e., MRC-1) and pro-inflammatory (i.e., IL6 and TNFα) markers compared to bare and non-doped biomimetic NPs.

Similar strategies have also been employed in the use of peripheral blood mononuclear cells (PBMCs) as a promising source of cell membrane for NP coating. Compared to conventional NPs, PBMC-derived biomimetic nanoparticles are endowed with leukocyte properties such as immune evasion, longer blood circulation, and tumor recognition and targeting. As such, several attempts have been made to develop immune cell-derived NPs. For example, Cao et al. cloaked drug-carrying liposomes with cell membranes isolated from macrophages for targeting metastatic lungs sites in a 4T1 breast cancer mouse model. These membrane-decorated emtansine liposomes (MELs) were endowed with enhanced uptake in metastatic cells resulting in inhibitory effects on cell viability. Furthermore, MELs exhibited specific metastatic-targeting potential in vivo with anti-metastatic activity observed in a metastatic breast cancer mouse model [62]. Based on a similar strategy, Kang et al. demonstrated the efficacy of neutrophil-mimetic nanoparticles loaded with proteasome inhibitor, carfilzomib, in selectively targeting CTCs both in blood and pre-metastatic niche [63]. Referred to as NM-NP-CFZs, these biomimetic NPs exhibited an enhanced in vitro binding ability to 4T1 breast cancer cells along with higher CTC association in vivo and improved homing to the pre-metastatic niche compared to uncoated NPs, thereby resulting in a decrease in the formation of tumor nodules.

## Conclusions

**Fig. 2:**
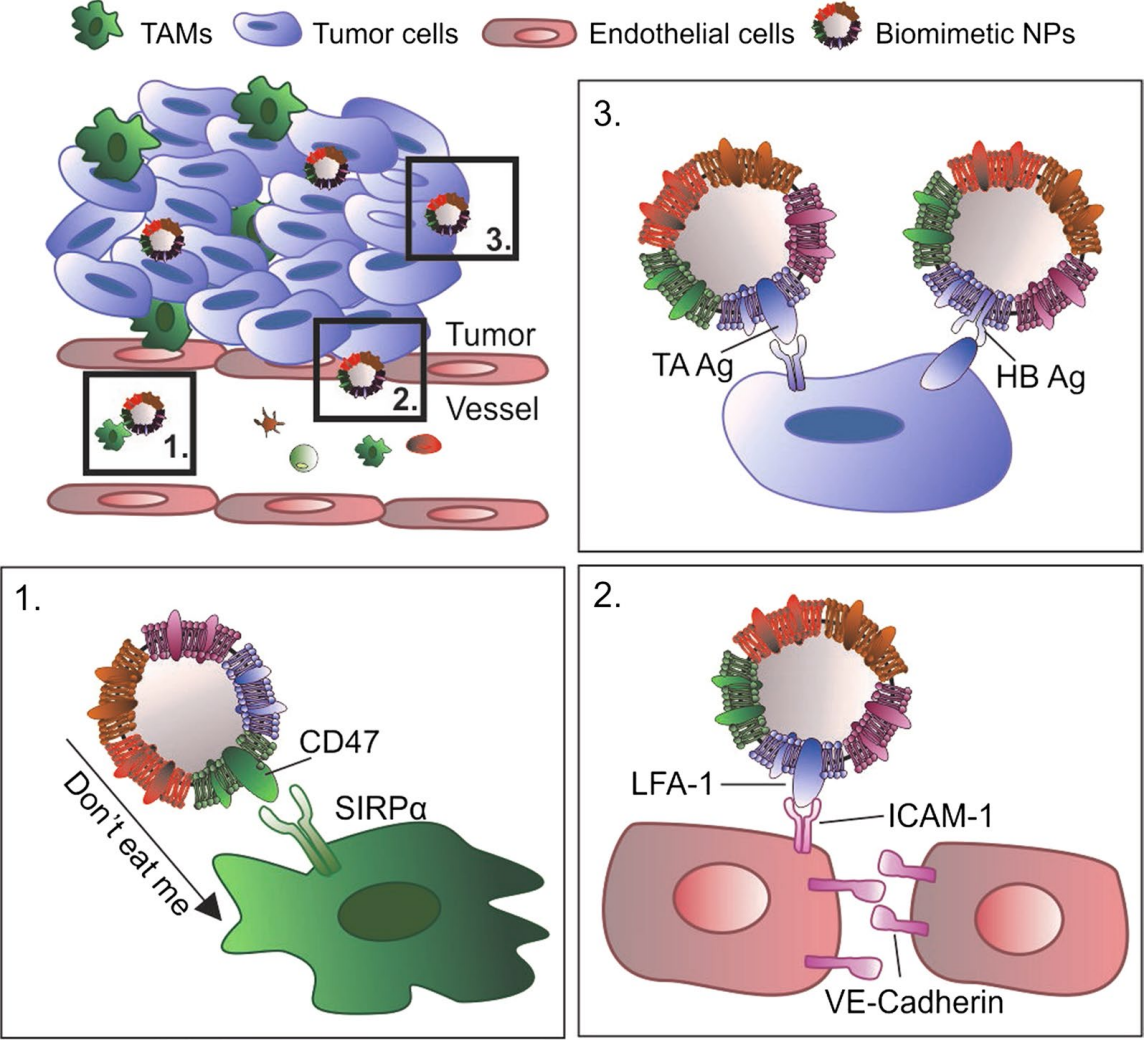
Biomimetic nanoparticles (NPs) inherited specific properties from the donor cell source, such as: 1. ability to escape mononuclear phagocyte system (MPS) resulting from the expression of the “do not eat me” cell signal, CD47, and its interaction with its receptor signal-regulatory protein alpha (SIRPα) on the macrophage surface; 2. adhesion to inflamed endothelium and activation of the Lymphocyte function-associated antigen 1/Intracellular adhesion molecule-1 (LFA-1/ICAM-1) pathway that alters tight junctions between endothelial cells, resulting in increased vessel permeability; 3. tumor targeting potential via expression of tumor associated (TA) and homotypic binding (HB) antigens (Ag) recognized by tumor cells. *TAMs* Tumor associated macrophages

The tumor microenvironment is composed of tumor cells surrounded by a variety of additional cells (i.e., fibroblast, immune and stromal cells) that participate in the cell-signaling network to create a neoplastic niche while supporting tumor growth and metastasis [64, 65]. As such, disruption of the cross-talk occurring in this microenvironment could potentially minimize tumor cell proliferation, leading to a reduction in aggressiveness that favors increased therapeutic potency [66]. Taking inspiration from the natural trafficking that occurs within the body, biomimetic NPs have been developed as a novel tool capable of interacting with the various cellular components within the tumor microenvironment [12], with the potential to interfere with cellular cross-talk. Indeed, beyond serving as simple drug carriers, biomimetic NPs are endowed with specific properties inherited from the donor cell source such as the ability to: (i) avoid macrophage phagocytosis; (ii) adhere to activated endothelia and (iii) preferentially accumulate at the tumor site (Fig. 2).

Altogether, the surface modification with membrane proteins provides biomimetic NPs with the ability to decrease opsonization and prolong circulation within the body, thus facilitating NP targeting. More recently, researchers have begun to explore combining proteins extracted from multiple cell sources as a strategy to develop biomimetic NPs with multiple functions and overall greater therapeutic efficacy (Fig. 1). Dehaini et al. pioneered this approach coating NPs with both RBC and platelet membranes. This chimeric formulation presented a higher systemic circulation and enhanced tumor cell targeting [67]. A potential risk that could arise from prolonged circulation time and avoidance of filtering organs (e.g., liver, spleen) is the non-specific targeting of NPs in tissues and organs where they could elicit potential toxicity. In general, we expect biomimetic NPs to be less toxic compared to inorganic and polymeric NPs due to their cell-derived elements. Indeed, the clearance of biomimetic NPs follows the physiological clearance of protein and lipid organic components. To date, our work has demonstrated that biomimetic NPs present decreased liver sequestration as well as decreased lung and spleen accumulation [59] with reduction in systemic toxicity [56].

Another important player in biomimetic NP function is the protein corona (PC): a layer of biomolecules that binds on the surface of NPs when dispersed in biological fluids. Contrarily to uncoated NPs, the presence of proteins on the biomimetic NP’s surface could favor the adsorption of specific proteins over others. For example, leukocyte membrane proteins in the liposome bilayer affect the adsorption of blood soluble proteins in the corona. We studied in vivo the evolution of the PC over time and revealed that the integration of leukocyte membrane proteins into the leukosome bilayer influenced the number, amount, orientation and type of plasma proteins adsorbed by the NPs, thus affecting NP biodistribution and interaction with cells [56].

However, despite the significant progress made in the development of biomimetic strategies, some aspects still needed further investigation to translate these NPs into a clinically-viable tool. To minimize immunoreactivity, biomimetic NPs could be ideally fabricated from the cells of the patient to be treated. However, due to the limited number of some cell subpopulations within the body, an ex vivo amplification step would be necessary upon isolation of the right cell types. Consequently, there is the need to create standard protocols to guarantee reproducibility, test batch-to-batch properties and achieve consistent systemic effects in vivo. While more studies need to be performed en route to clinical translation, experimental evidence suggests that biomimetic NPs could provide a solution to overcome major limitations and drawbacks of previous generations of NPs and could represent a promising tool in the treatment of cancer.

## Abbreviations

NPs: nanoparticles
NGs: nanoghosts
RBCs: red blood cells
PEG: polyethylene glycol
DOX: doxorubicin
TRAIL: tumor necrosis factor (TNF)-related apoptosis inducing ligand
CTCs: circulating tumor cells
CSCs: cancer stem cells
MSCs: mesenchymal stem cells
MPS: mononuclear phagocyte system
